# RETRACTION: PCAT‐1′ s Role in Wound Healing Impairment: Mitochondrial Dysfunction and Bone Marrow Stem Cell Differentiation Inhibition via PKM2/β‐Catenin Pathway and its Impact on Implant Osseo‐Integration

**DOI:** 10.1111/iwj.70484

**Published:** 2025-04-02

## Abstract

**RETRACTION:** DengT. , LiuQ. , LiY. , ZhuX. , LongY. , LiuB. , PangJ. , and ZhaoL. , “PCAT‐1′ s Role in Wound Healing Impairment: Mitochondrial Dysfunction and Bone Marrow Stem Cell Differentiation Inhibition via PKM2/β‐Catenin Pathway and its Impact on Implant Osseo‐Integration,” International Wound Journal 21, no. 4 (2024): e14589, 10.1111/iwj.14589.38135901 PMC10961899

The above article, published online on 22 December 2023, in Wiley Online Library (http://onlinelibrary.wiley.com/), has been retracted by agreement between the journal Editor in Chief, Professor Keith Harding; and John Wiley & Sons Ltd. Following an investigation by the publisher, all parties have concluded that this article was accepted solely on the basis of a compromised peer review process. The editors have therefore decided to retract the article. The authors did not respond to our notice regarding the retraction.

**RETRACTION:** T. Deng , Q. Liu , Y. Li , X. Zhu , Y. Long , B. Liu , J. Pang , and L. Zhao , “PCAT‐1′ s Role in Wound Healing Impairment: Mitochondrial Dysfunction and Bone Marrow Stem Cell Differentiation Inhibition via PKM2/β‐Catenin Pathway and its Impact on Implant Osseo‐Integration,” International Wound Journal 21, no. 4 (2024): e14589, 10.1111/iwj.14589.38135901 PMC10961899

The above article, published online on 22 December 2023, in Wiley Online Library (http://onlinelibrary.wiley.com/), has been retracted by agreement between the journal Editor in Chief, Professor Keith Harding; and John Wiley & Sons Ltd. Following an investigation by the publisher, all parties have concluded that this article was accepted solely on the basis of a compromised peer review process. The editors have therefore decided to retract the article. The authors did not respond to our notice regarding the retraction.

